# Sortilin, SorCS1b, and SorLA Vps10p sorting receptors, are novel γ-secretase substrates

**DOI:** 10.1186/1750-1326-1-3

**Published:** 2006-06-12

**Authors:** Andrew C Nyborg, Thomas B Ladd, Craig W Zwizinski, James J Lah, Todd E Golde

**Affiliations:** 1 Department of Neuroscience, Mayo Clinic Jacksonville, Mayo Clinic College of Medicine, 4500 San Pablo Road, Jacksonville, Florida 32224, USA; 2 Department of Neurology, Center for Neurodegenerative Disease, Emory University, Whitehead Biomedical Research Building, 615 Michael Street, Suite 505, Atlanta, GA 30322, USA

## Abstract

**Background:**

The mammalian Vps10p sorting receptor family is a group of 5 type I membrane homologs (Sortilin, SorLA, and SorCS1-3). These receptors bind various cargo proteins via their luminal Vps10p domains and have been shown to mediate a variety of intracellular sorting and trafficking functions. These proteins are highly expressed in the brain. SorLA has been shown to be down regulated in Alzheimer's disease brains, interact with ApoE, and modulate Aβ production. Sortilin has been shown to be part of proNGF mediated death signaling that results from a complex of Sortilin, p75^NTR ^and proNGF. We have investigated and provide evidence for γ-secretase cleavage of this family of proteins.

**Results:**

We provide evidence that these receptors are substrates for presenilin dependent γ-secretase cleavage. γ-Secretase cleavage of these sorting receptors is inhibited by γ-secretase inhibitors and does not occur in PS1/PS2 knockout cells. Like most γ-secretase substrates, we find that ectodomain shedding precedes γ-secretase cleavage. The ectodomain cleavage is inhibited by a metalloprotease inhibitor and activated by PMA suggesting that it is mediated by an α-secretase like cleavage.

**Conclusion:**

These data indicate that the α- and γ-secretase cleavages of the mammalian Vps10p sorting receptors occur in a fashion analogous to other known γ-secretase substrates, and could possibly regulate the biological functions of these proteins.

## Background

γ-Secretase is a multi-component protease complex comprised of Presenilin (PS) 1 or 2 with Aph-1, Pen-2, and Nicastrin [1,2] that cleaves type I membrane proteins within their transmembrane domains. γ-Secretase catalyzes a number of important physiological and pathophysiological cleavages. Following ectodomain cleavage of the amyloid precursor protein (APP) [3] by β-secretase, γ-secretase cleavage releases the amyloid beta peptide (Aβ) that accumulates in the brains of patients with Alzheimer's disease (AD) [4]. γ-Secretase also plays a key role in mediating signaling via the Notch receptors [5-7]. In most cases, knockout of presenilin or other components of the γ-secretase complex produces an embryonic lethal phenotype, that resembles the phenotype produced by knockout of Notch 1.

To date, more than 25 γ-secretase substrates have been identified. [8-28]. All identified γ-secretase substrates are type I transmembrane proteins [29] and contain a putative stop transfer sequence immediately following the transmembrane region [30]. In most cases, ectodomain shedding precedes intramembrane γ-secretase cleavage [31]. For a growing lists of substrates, γ-secretase cleavage has been shown to mediate downstream signaling events [32].

**Figure 1:**
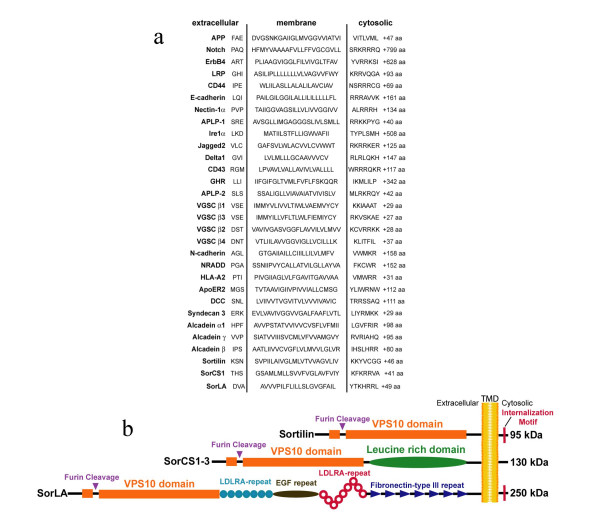
**Schematics of γ-secretase substrates and the mammalian Vps10p proteins**. **a) **Alignment of the transmembrane domains and juxtamembrane region of known γ-secretase substrates The number of additional amino acids in the cytoplasmic domain following the sequence shown is indicated by "+ aa". **b) **A schematic of the human Vps10p containing proteins is shown. Sortilin is a 95 kDa glycoprotein. SorCS1, 2, and 3 contain a leucine rich domain and are ~130 kDa. The largest of the homologs, SorLA is 250 kDa and contains a host of receptor binding domains including 14 different low density lipoprotein receptor sites, and EGF repeat, and a fribronectin type III repeat.

Although many proteases preferentially cleave at consensus sequences within the substrate, no consensus sequence for cleavage by γ-secretase has been identified. γ-Secretase cleavage always occurs within a putative hydrophobic transmembrane region, but a variety of different sites are cleaved even in a single substrate. Alignment of γ-secretase substrate transmembrane domains provides little insight into the sequence requirements for proteolysis to occur (Figure 1a) [33]. Likewise mutations of cleavage sites have provided little definitive information on the nature of the cleavage, though several models have been proposed [34].

Following ectodomain shedding, γ-secretase cleavage liberates both the cytoplasmic fragment and a small secreted peptide. For several substrates the liberated cytoplasmic domain has been shown to translocate to the nucleus where it is involved in nuclear signaling (Notch ErbB4, Delta-1 Jagged, APLP1/2). This process is more generally referred to as regulated intramembrane proteolysis (RIP). RIP of Notch has been intensively studied. Ligand binding to the Notch extracellular domain results in ectodomain cleavage, which is then followed by γ-secretase cleavage. Once liberated the notch intracellular domain (NICD) translocates to the nucleus where it binds to CSL family of transcription factors [35,36]. Notch binding to CSL has been shown to convert CSL from a transcriptional repressor to a transcriptional activator [5]. Analogously nuclear signaling of the CD44 [37,38] and N-cadherin [39] cytoplasmic domains following γ-secretase cleavage is indirectly achieved through CBP (CREB-binding protein) activation or suppression, respectively. In some cases nuclear translocation of the cytoplasmic domain is not required for signaling following γ-secretase cleavage [8,13,14,40]. Not all γ-secretase substrates appear to undergo RIP, as ligand binding is not necessary for the initiation of cleavage. For example, APP does not appear to require ligand association in order to initiate ectodomain shedding which occurs prior to γ-secretase cleavage.

In most cases, signaling initiated by γ-secretase cleavage appears to be an activation event. However, cleavage of the substrate deleted in colorectal cancer (DCC) attenuates receptor-mediated intracellular signaling pathways that are critical in regulating glutamatergic synaptic transmission and memory processes [18,41].

Because of their topologic similarity to other γ-secretase substrates and evidence for ectodomain shedding [42] (Figure 1a), we hypothesized that the mammalian Vps10p containing family of proteins might be γ-secretase substrates. Sortilin, SorLA and SorCS1, 2, and 3 comprise the five identified mammalian Vps10p sorting receptors and have a number of features in common. First, all are type I membrane proteins (Figure 1b). Second, all contain a luminal/extracellular cysteine-rich Vps10p domain homologous to the binding domain of the yeast sorting receptor for carboxypeptidase Y. Third, all contain a putative furin cleavage site. SorCS1-3 and SorLA also contain additional extracellular domains thought to be involved in ligand binding (Figure 1b). Sortilin is also known as neurotensin receptor 3 or gp95, and SorLA is often called LR11. These receptors are hypothesized to have pleiotropic functions in chaperoning and targeting various cargoes bound to their luminal Vps10p domains between various intracellular organelles [43,44].

These sorting receptors are expressed at high levels in the CNS and in neurons [44-46]. Sortilin is part of the machinery that governs cell survival in developing neuronal tissue and a key determinant in the induction of posttraumatic neuronal apoptosis [47]. It also mediates rapid endocytosis of lipoprotein lipase [48], neurotensin [49], and the proform of nerve growth factor [47]. Sortilin has been shown to target proteins in the Golgi for transport to late endosomes. Of its many sorting and signaling functions, Sortilin was shown to play a role in p75^NTR ^"death signaling". The cell death signal is a result of a complex of p75^NTR^, Sortilin and the precursor form of nerve growth factor (proNGF) [47] or pro brain-derived neurotrophic factor [50].

Recent data have suggested a role for SorLA in AD. SorLA was shown to be a receptor for, and interact with, ApoE [51-54]. In addition, it is reduced in AD brains versus age matched controls, interacts with APP [55] and regulates Aβ production [56,57].

we provide evidence that Sortilin, SorCS1b and SorLA are sequentially cleaved by an α-secretase like activity followed by γ-secretase. α-Secretase cleavage results in secretion of a large extracellular domain of the Vps10p substrates and γ-secretase liberates a COOH-terminal fragment (CTF) from the membrane. These data extend very recent studies demonstrating that Sortilin, SorCS1, SorCS2, SorCS3, and SorLA undergo ectodomain shedding [42] and that SorLA can undergo γ-secretase cleavage [28].

## Results

### Evidence for sequential α- and γ-secretase cleavage of Sortilin

**Figure 2:**
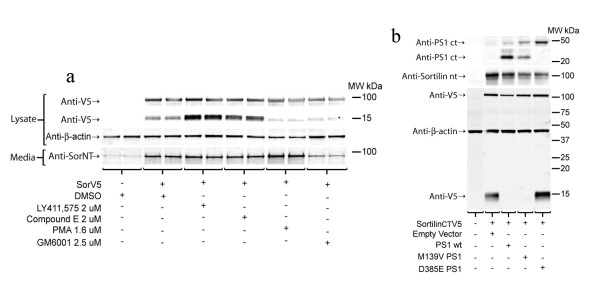
**Sortilin is processed into a CTF that is increased by γ-secretase inhibition or knockout**. **a) **HEK 293T cells transiently transfected with sorV5 produce a ~95 kDa holoprotein labeled by anti-V5 and anti-Sor NT antibody (not shown). A ~16 kDa CTF of SorV5 is detected by an anti-V5 antibody is present in the cell lysate, and anti-SorNT detects a secreted fragment of ~95 kDa (sSor) that does not label with anti-V5. The anti-V5 positive SorV5 CTF increased with γ-secretase inhibitor treatment (LY411,575 and compound E) and decreased with either PMA or a metalloprotease inhibitor, GM6001. sSor increased upon PMA treatment and decreased upon treatment with a metalloprotease inhibitor, GM6001. **b) **Mouse embryonic fibroblasts that are PS^-/- ^were transiently transfected with SorV5 plus empty vector, PS1, PS1 M139V, or PS1 D385E. In the absence of PS1 or the presence of a dominant negative PS1 mutant (D385E) the levels 16 kDa SorV5 CTF are markedly increased. However, cotransfection of PS1 wt or PS1 familial Alzheimer's disease linked mutant (M139V) and SorV5 lead to an almost complete reduction in the levels of 16 kDa anti-V5 positive SorV5 CTF. Anti-β-actin was included as a loading control and anti-PS1ct antibody demonstrated that PS1 wt and M139V underwent endoproteolysis whereas D385E did not.

HEK 293T cells were transiently transfected with Sortilin containing a V5 epitope tag at the COOH-terminus (SorV5) and treated with a variety of inhibitors or PMA. The epitope tagged SorV5 construct was used because we have been unable to obtain a reliable anti-sortilin COOH-terminal antibody. Cell lysates and media from the transiently transfected cells were analyzed by SDS PAGE and Western blotting. Consistent with previous studies showing that the untagged Sortilin holoprotein is a ~95 kDa glycoprotein [58], transfection of the SorV5 resulted in expression of a 95 kDa protein that was detected both by an anti-Sortilin NH2-terminal antibody (not shown) and anti-V5 (Figure 2a). In addition, a SorV5 CTF was detected with the anti-V5 antibody at ~16 kDa (Figure 2a). This band was not detected by the NH2-terminal Sortilin antibody (not shown). γ-Secretase inhibitor treatment markedly increased the ~16 kDa SorV5 CTF in cell lysates of cells transiently overexpressing SorV5 (Figure 2a), but did not appreciably alter the amount of soluble Sortilin (sSor) that was detected in the media with an anti-Sortilin NH2-terminal antibody but not anti-V5 (Figure 2a). Activation of protein kinase C by PMA has been shown to enhance ectodomain shedding of APP by α-secretase [59-62] and resulted in an increase in sSor detected in the media (Figure 2a). GM6001, a broad spectrum hydroxamic acid based inhibitor of matrixmetalloproteases and disintegrin-metalloproteases [63], has been shown to inhibit α-secretase cleavage of APP and decreased sSor levels detected in the media. Both GM6001 and PMA treatment decreased total levels of the 16 kDa SorV5 CTF (Figure 2a). Anti-β-actin was used to demonstrate loading consistency between wells.

The increase in 16 kDa sorV5 CTF resulting from γ-secretase inhibition suggested that the SorV5 CTF was a precursor to γ-secretase cleavage. We then expressed SorV5 in cells lacking PS1 and PS2. Transfection of SorV5 into PS^-/- ^MEF cells resulted in an accumulation of the 16 kDa CTF (Figure 2b). Cotransfection of PS^-/- ^MEF with SorV5 and either PS1 wt or a familial Alzheimer's disease linked mutant PS1 (M139V) caused the 16 kDa SorV5 CTF band to almost completely disappear (Figure 2b). Cotransfection of a dominant negative PS1 aspartate mutant (D385E) did not reduce levels of the 16 kDa SorV5 CTF. As expected, PS1 wt and M139V PS1 expression resulted in generation of endoproteolyzed PS1 CTF (Figure 2b); and expression of PS1 D385E resulted in accumulation of the PS1 holo protein (Figure 2b).

### γ-secretase processing of SorV5 CTF in lipid rafts

**Figure 3:**
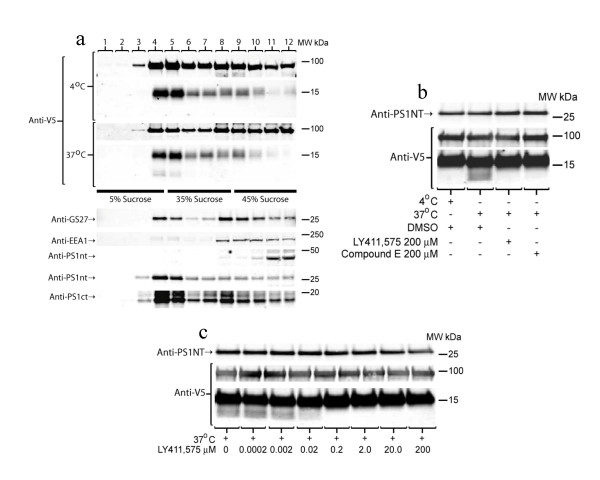
**Sucrose gradient fractionation of SorV5 overexpressing cells demonstrates enrichment of the 16 kDa CTF in buoyant "lipid raft" fractions and generation of a smaller SorV5 CTF**. **a) **HEK cells stably overexpressing sorV5 were sucrose gradient fractionated and 1 ml fractions were collected from the top. Each fraction was analyzed by SDS PAGE on 12% XT Bis-Tris SDS PAGE (MES Buffer) and Western blotting. "4°C" were samples that were maintained at 4°C until SDS PAGE loading buffer was added. "37°C" indicates samples that were incubated at 37°C for 2 hours and then prepared as western samples. A smaller SorV5 CTF was detected in fractions 4 and 5 of the 37°C incubated sample. Anti-V5 antibody detected the SorV5 holo protein at 95 kDa, a CTF at 16 kDa and a smaller CTF in the "37°C" lanes 4 and 5. Anti-GS27 was used as a lipid raft marker (fractions 4–5), Anti-EEA1 is an early endosomal marker that was not found in lipid raft. PS1 CTF and NTF were enriched in raft fractions 4 and 5. **b) **HEK cells stably overexpressing sorV5 were pretreated with a γ-secretase inhibitor (IL-aldehyde) for 30 hours and sucrose gradient fractions 4, 5 were combined and analyzed for generation of the smaller SorV5 CTF. Samples were equally divided and incubated at 37°C (4°C for control) in the absence or presence of γ-secretase inhibitors, LY411575, and Compound E. A smaller SorV5 CTF band was detected as a result of incubation at 37°C but was blocked by γ-secretase inhibitors. **c) **Samples were prepared as in b). Generation of the smaller SorV5 CTF was inhibited in a dose dependant fashion by a γ-secretase inhibitor, LY411,575. Anti-PS1NT demonstrates the loading consistency.

In figure 2b the 16 kDa CTF, a putative γ-secretase substrate, increased when no PS was present. Under conditions amenable to γ-secretase cleavage, the 16 kDa band diminished but a smaller product band was not detected. It has been demonstrated that γ-secretase activity is enriched by sucrose gradient fractionation into cholesterol rich microdomains called lipid rafts [64-67]. To further confirm that the 16 kDa SorV5 CTF is a γ-secretase substrate, cells stably overexpressing SorV5 were lysed in 2% CHAPSO to maintain the γ-secretase complex and sucrose gradient fractionated as described in the Methods. Fractions were analyzed by SDS PAGE and Western blotting (Figure 3a). As previously shown, PS1 CTF and NH2-terminal fragment (NTF) were enriched in the buoyant fractions 4 and 5 whereas holo PS1 was primarily found in more dense fractions 10, 11 and 12 (figure 3a). GS27, a known lipid raft marker, was enriched in the buoyant fractions as shown previously [64]. However, EEA1, an early endosomal marker, was found primarily in dense fractions. The 95 kDa holo SorV5 fractionated to both the buoyant and dense fractions much like holo APP [64]. A SorV5 CTF (~16 kDa) was enriched in the buoyant factions (Figure 3a, "4°C"). Incubation of the all fractions at 37°C for two hours generated a smaller SorV5 CTF (~13 kDa) that was only detected in buoyant fractions (Figure 3 "37°C"). These data provide evidence that the lipid raft portion of the sucrose gradient contained all the components necessary to generate a smaller SorV5 CTF upon incubation at 37°C.

Having observed the *in vitro *generation of a smaller SorV5 CTF in fractions 4 and 5 as a result of incubating at 37°C for 2 hours, we repeated the experiment with cells that had been pretreated with 50 μM IL-aldehyde, a γ-secretase inhibitor. These cells were lysed and sucrose gradient fractionated. Buoyant fractions 4 and 5 were combined and equal aliquots were incubated at 37°C for two hours plus and minus γ-secretase inhibitors. During the same period a negative control was incubated at 4°C for two hours. As shown in Figure 3a, when the buoyant fractions were incubated at 37°C for two hours a smaller SorV5 CTF band was generated (figure 3b DMSO). In the sample that remained at 4°C during the incubation period or those incubated in the presence of γ-secretase inhibitors no smaller SorV5 CTF was detected (Figure 3b). Furthermore, generation of the smaller SorV5 CTF was inhibited by another γ-secretase inhibitor (LY411,575) in a dose dependant fashion (Figure 3c). Anti-PS1NT antibody was used to demonstrate loading consistency.

### SorCS1b, evidence for α and γ-secretase processing of a second mammalian Vps10p

**Figure 4:**
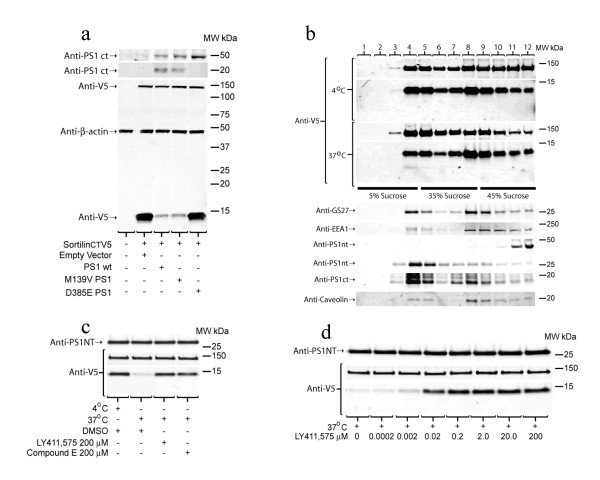
**PS dependent γ-secretase cleavage of SorCS1bV5 in lipid rafts**. **a) **Mouse embryonic fibroblasts that are PS^-/- ^were transiently transfected with SorCS1bV5 in the same fashion as figure 3a. Cells transiently transfected with sorCS1bV5 produce a ~130 kDa holoprotein labeled by anti-V5. In the absence of PS1 or the presence of a dominant negative PS1 mutant (D385E) the levels 13 kDa SorCS1bV5 CTF are markedly increased. However, cotransfection of PS1 wt or PS1 familial Alzheimer's disease linked mutant (M139V) and SorCS1bV5 lead to a reduction in the levels of 13 kDa anti-V5 positive SorCS1bV5 CTF. Anti-β-actin was included as a loading control and anti-PS1ct antibody demonstrated that PS1 wt and M139V underwent endoproteolysis whereas D385E did not. **b) **HEK cells stably overexpressing sorCS1bV5 were sucrose gradient fractionated and 1 ml fractions were collected from the top. Each fraction was analyzed by SDS PAGE on 12% XT Bis-Tris SDS PAGE (MES Buffer) and Western blotting. "4°C" were samples that were maintained at 4°C until SDS PAGE loading buffer was added. "37°C" indicates samples that were incubated at 37°C for 2 hours and then prepared as western samples. A faint SorCS1bV5 CTF was detected in fractions 4 and 5 of the 37°C incubated sample. Anti-V5 antibody detected the SorV5 holo protein at 130 kDa, a CTF at 13 kDa and a smaller CTF in the "37°C" lanes 4 and 5. Anti-GS27 was used as a lipid raft marker (fractions 4–5), Anti-EEA1 is an early endosomal marker that was not found in lipid raft. PS1 CTF and NTF were enriched in raft fractions 4 and 5. **c) **HEK cells stably overexpressing sorCS1bV5 were pretreated with a γ-secretase inhibitor (IL-aldehyde) for 30 hours and sucrose gradient fractions 4, 5 were combined and analyzed for generation of the smaller SorCS1bV5 CTF. Samples were equally divided and incubated at 37°C (4°C for control) in the absence or presence of γ-secretase inhibitors, LY411575, and Compound E. A smaller SorV5 CTF band was not detected as a result of incubation at 37°C but the disappearance of the 13 kDa SorCS1bV5 CTF was blocked by γ-secretase inhibitors. **d) **Samples were prepared as in b). Disappearance of the SorCS1bV5 CTF was inhibited in a dose dependant fashion by a γ-secretase inhibitor, LY411,575. Anti-PS1NT demonstrates the loading consistency.

To further establish that the mammalian Vps10p containing family members are processed by α and γ-secretase similar experiments were performed with SorCS1b tagged with V5 at the COOH-terminus (SorCS1bV5). Consistent with previous studies showing that the untagged SorCS1b or His tagged holoprotein was a ~130 kDa [68], transfection of the SorCS1bV5 resulted in expression of a 130 kDa protein that was detected by anti-V5 (figure 4a). Transfection of SorCS1bV5 into PS^-/- ^MEF cells resulted in an increase in the 13 kDa CTF (Figure 4a). Cotransfection of PS^-/- ^MEF with SorCS1bV5 and either PS1 wt or a familial Alzheimer's disease linked mutant PS1 (M139V) caused a decrease in the 13 kDa SorV5 CTF band (Figure 4a). Cotransfection of a dominant negative PS1 aspartate mutant (D385E) did not reduce levels of the 13 kDa SorCS1bV5 CTF. Sucrose gradient fractionation of a cell lysate from HEK cells stably overexpressing SorCS1bV5 lead to an enrichment of a ~13 kDa SorCS1bV5 CTF in buoyant fractions at 4 and 5 (Figure 4b). Incubating the fractions at 37°C for two hours generated a faintly detectable anti-V5 positive band at ~10 kDa. More consistent than the detection of this band, was a decrease in the 13 kDa SorCS1bV5 CTF band observed after incubation at 37°C for two hours (Figure 4c and 4d). The decrease in the ~13 kDa SorCS1bV5 band was inhibited by both Compound E (Figure 4c) and LY411,575 in a concentration dependent fashion (Figure 4d). Detection of the soluble SorSC1b NH2-terminus in the media was not possible due to the lack of a reliable NH2-terminal anti-SorCS1 antibody.

### PS dependant SorLA processing

**Figure 5:**
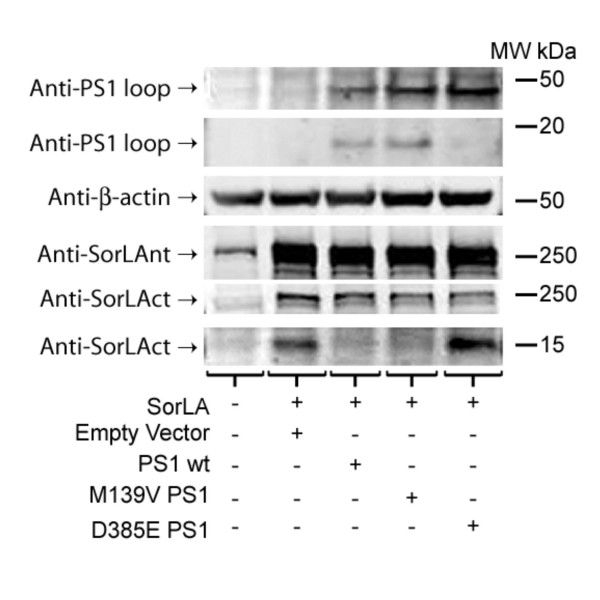
**PS dependent cleavage of SorLA CTF**. Mouse embryonic fibroblasts that are PS^-/- ^were transiently transfected with SorLA in the same fashion as figure 3a. Cells transiently transfected with SorLA produce a ~250 kDa holoprotein labeled by anti-V5. In the absence of PS1 or the presence of a dominant negative PS1 mutant (D385E) the levels 15 kDa anti-SorLAct positive SorLA CTF markedly increased. Cotransfection of PS1 wt or PS1 familial Alzheimer's disease linked mutant (M139V) and SorLA wt lead to a reduction in the levels of 15 kDa anti-SorLA positive SorLA CTF. Anti-β-actin was included as a loading control and anti-PS1ct antibody demonstrated that PS1 wt and M139V underwent endoproteolysis whereas D385E did not.

Using a wt SorLA construct and a COOH-terminal anti-SorLA antibody (anti-SorLAct) we have obtained evidence that that SorLA is a γ-secretase substrate as well. The SorLA holoprotein was detected by the SorLA COOH-terminal antibody at 250 kDa (Figure 5) consistent with previous reports [69]. Transfection of SorLA into PS^-/- ^MEF cells resulted in accumulation of a 15 kDa SorLA CTF detected by an anti-SorLAct antibody (Figure 5). However, cotransfections with SorLA and either PS1 wt or an familial Alzheimer's disease linked mutant PS1 (M139V) caused a decrease in the 15 kDa SorLA CTF (Figure 5). Cotransfection of sorLA with a dominant negative PS1 aspartate mutant (D385E) did not alter levels of the 15 kDa SorLA CTF.

## Discussion

To date, more than 25 γ-secretase substrates have been identified (Figure 1a). γ-Secretase cleavage of certain substrates mediates normal physiologic signaling. Given the growing list of substrates, it has been postulated that γ-secretase may function much like the proteosome of the membrane assisting in the degradation of type I transmembrane proteins by liberating them from the membrane [70]. This notion may be supported by the promiscuity with which γ-secretase cleaves multiple substrates at a variety of different sites (Figure 1a). It is possible that overexpression of type I transmembrane proteins may result in non-physiologic γ-secretase cleavage. However, to date, all γ-secretase substrates initially identified using overexpression studies in eukaryotic cells have proven to be authentic endogenous substrates. Indeed, numerous γ-secretase substrates like APP, Notch, and CD44 have withstood the scrutiny of genetic, biochemical, animal model data that establish both their authenticity as true γ-secretase substrates and the physiologic relevance of γ-secretase cleavage.

Sortilin, SorCS1b and SorLA are all members of the mammalian Vps10p sorting receptor family. In this manuscript we provide several lines of evidence that these proteins are γ-secretase substrates. We detect truncated CTF derived from COOH-terminally V5 epitope tagged Sortilin and SorCS1b and untagged SorLA. These CTF appear to be derived from the holoproteins by an α-secretase like activity. γ-Secretase inhibitors and expression of the proteins in presenilin knockout cells results in marked accumulation of these CTF, a phenomena reversed by expression wt or FAD-linked mutant PS1. These CTF are enriched in buoyant lipid raft fractions where γ-secretase activity resides. Incubation of SorV5 or SorCS1bV5 CTF containing raft fractions leads to generation of a smaller cleavage product in the case of Sortilin and a decrease of the CTF in the case of SorCS1bV5. The generation of the smaller SorV5 CTF and the decrease in SorCS1bV5 CTF are inhibited by a γ-secretase inhibitor. Collectively these data indicate that γ-secretase has the capacity to cleave overexpressed Sortilin, SorCS1b and SorLA.

During the preparation of this manuscript evidence was reported that SorLA was a γ-secretase substrate [28]. Bohm et al demonstrated that Myc tagged SorLA underwent PS dependent γ-secretase processing and that following γ-secretase cleavage the cytoplasmic portion was found in the nucleus [28]. Combined with our data, demonstrating that Sortilin, SorCS1b, and SorLa are γ-secretase substrates, these data provide evidence that the entire family of mammalian Vps10p containing type I proteins may contain important biologically active signaling peptides in their COOH-termini.

Studies showing intramembrane γ-secretase cleavage of endogenous Vps10p sorting receptor will be needed to show that these proteins are in fact authentic γ-secretase substrates. As noted before it is unlikely that the overexpression studies performed here will be misleading. Given the complex trafficking, sorting, and signaling functions mediated by mammalian Vps10p sorting receptors it will be interesting to determine whether γ-secretase cleavage regulates the normal function of these proteins. Indeed, it may be that some of the trafficking deficits in PS deficient cells could be attributed to the lack of γ-secretase cleavage of several Vps10p proteins. Though speculative there is some evidence that Vps10p receptors may play a role in AD and in neuronal death. SorLA has been shown to be down regulated in AD, and plays a role in APP trafficking and Aβ production [56,71]. Neuronal death signaling was shown to result from a complex of two γ-substrates, Sortilin and p75^NTR ^[13,14], in conjunction with proNGF or proBDNF [47]. Additional studies are needed to clarify the physiologic and possible pathologic role of γ-secretase cleavage of mammalian Vps10p sorting receptors.

## Materials and methods

### DNA constructs

Full length Sortilin and SorCS1b plasmid constructs were purchased from Origene. A V5 epitope was cloned into the pCMV6-XL plasmid at the COOH-terminus immediately preceding the stop codon (SorV5 and SorCS1V5). SorLA wt plasmid was described previously [57]. PS1 wt, M139V, and D385E were described previously [34]. All constructs were verified by sequencing.

### DNA transfection of PS^-/- ^MEF cells

Mouse embryonic fibroblasts (MEF) deficient in both PS1 and PS2 (PS^-/-^) were characterized previously [72]. Efficient transfection of these cells was achieved using the Amaxa nucleofector system. Briefly, using the Amaxa MEF kit 2, 3 μg of DNA and 3 × 10^6 cells/reaction the transfection was performed with the "O-05" mouse neuron program. Transfected cells were plated on 10 cm plate with 8 ml of growth media. Cells were harvested after 24 hours.

### SorV5 and SorCS1bV5 stable cell lines and culture

Human embryonic kidney (HEK) 293 cells were transfected in reduced serum Opti-Mem (Gibco) with 2 μg of DNA and 8 μl of Fugene. Cells were incubated for 6 hours and then allowed to equilibrate in standard growth media (DMEM 2% fetal bovine serum, 8% normal calf serum, 1% penicillin streptomycin) for 24 hours. Selection antibiotic was then added to the cells and maintained throughout the experiments. Transient expression experiments were performed the same but with HEK 293T cells.

Cells treated with inhibitors were incubated for 18 hours using the concentration reported and 1% DMSO. phorbol 12-myristate-13-acetate (PMA) treatment was performed for 4 hours.

### γ-secretase inhibitors

Inhibitors were all generated by the Mayo Clinic Chemistry core using published methods for each. All compounds were verified by NMR and Mass Spectroscopy.

### Lysis, antibodies, and Western blotting

Cells were lysed in 1% triton × 100 with 1× complete protease inhibitor (Roche) unless otherwise stated. Cell lysates were then spun at 14,000 RPM for 2 minutes to remove nuclei. BioRad XT loading buffer with reducing solution was added to each sample. SDS-PAGE was performed using BioRad Criterion gel system. 12% Bis-Tris XT gels were used unless otherwise stated with BioRad MES buffer. Gels were transferred to Millipore low-fluor PVDF for 90 minutes and 160 volts. Membranes were blocked in caesine (0.25%) and phosphate buffered saline solution and primary antibodies were used at the reported concentration in the blocking solution overnight at 4°C. Anti-V5 (Invitrogen) and anti-β-actin antibody (Sigma) antibodies were used at 1:1000. The anti-PS1 NTF (A4 from Dr. Paul Fraser) antibody I think) and anti-PS1 CTF (490) were used at 1:1000. The anti-SorLAct antibody was used at 1:500 [57]. Fluorescent antibodies containing either the 680 or 800 fluorophore were incubated with the membrane for 1 hour at room temperature at 1:20,000. Fluorescently labeled protein detection was performed using the Odyssey Scanner.

### Gradient fractionation

Sucrose gradients were run as described previously [64]. Briefly, cells were washed with 5 mL of ice cold PBS (pH 7.4) and lysed in 2.5 mL of 2% CHAPSO 0.15 M Na Citrate (pH 7.0) with 1× protease inhibitor cocktail (complete PI, Roche). The cleared lysate was then sequentially diluted with sucrose containing 0.15 M Na Citrate (pH 7.0) so that the final concentration of CHAPSO was 0.25% and sucrose was 45%. Four ml of this homogenate was then applied to the bottom of the centrifuge tube, and sequentially overlaid with 4 ml of 0.15 M Na Citrate (pH 7.0), 35% sucrose, 0.25% CHAPSO followed by 4 ml of 0.15 M Na Citrate (pH 7.0), 5% sucrose, 0.25% CHAPSO. The gradient was centrifuged for 19 hrs at 39,000 rpm in an SW-41 Ti rotor (Beckman) at 4°C. Following centrifugation, 1 mL fractions were collected from the top of the gradient.

## Abbreviations

Abbreviations: CTF, COOH-terminal fragment; NTF, NH2-terminal fragment; SorV5, sortilin that contain a COOH-terminal V5 his epitope tag; SorCS1bV5, SorCs1b that contain a COOH-terminal V5 his epitope tag; PS, presenilin; APP, amyloid precursor protein; AD, Alzheimer's disease; RIP, regulated intramembrane proteolysis; NICD, Notch intracellular domain; CBP, CREBP binding protein; DCC, deleted in colorectal cancer protein; PMA, phorbol 12-myristate-13-acetate; anti-SorLA, antibody specific to the COOH-terminus of SorLA; MEF, mouse embryonic fibroblasts; proNGF, proform of nerve growth factor; sSor, soluble NH2-terminal fragment of Sortilin; HEK, human embryonic kidney cell line; PS^-/-^, MEF cells that PS1 and PS2 are knocked out

## Authors' contributions

ACN and TEG contributed to the conception, design, analysis and interpretation of the data and were responsible to the manuscript preparation. ACN, TBL and CWZ carried out the experiments in this manuscript. JJL provided critical reagents for these experiments. ACN, TEG, and JJL all contributed to the interpretation and analysis of data. All authors read and approved the final manuscript.
